# RETRACTED: El-Masry et al. Potential Antitumor Activity of Combined Lycopene and Sorafenib Against Solid Ehrlich Carcinoma via Targeting Autophagy and Apoptosis and Suppressing Proliferation. *Pharmaceuticals* 2024, *17*, 527

**DOI:** 10.3390/ph19010062

**Published:** 2025-12-29

**Authors:** Thanaa A. El-Masry, Maysa M. F. El-Nagar, Nageh A. El Mahdy, Fatemah A. Alherz, Reham Taher, Enass Y. Osman

**Affiliations:** 1 Department of Pharmacology and Toxicology, Faculty of Pharmacy, Tanta University, Tanta 31527, Egypt; 2 Department of Pharmaceutical Sciences, College of Pharmacy, Princess Nourah bint Abdulrahman University, P.O. Box 84428, Riyadh 11671, Saudi Arabia

The journal retracts the article “Potential Antitumor Activity of Combined Lycopene and Sorafenib against Solid Ehrlich Carcinoma via Targeting Autophagy and Apoptosis and Suppressing Proliferation” [1] cited above.

Following publication, concerns were brought to the attention of the Editorial Office regarding the integrity of a number of images presented in this publication [1].

Adhering to our standard procedure, an investigation was conducted by the Editorial Office and Editorial Board that identified irregularities and partial duplication in Figures 2–4 presented in this article. While the authors collaborated with this process, full raw material meeting the journal’s requirements for original images (https://www.mdpi.com/journal/pharmaceuticals/instructions#oriimages, accessed on 3 December 2025) could not be provided for evaluation by the Editorial Board. Consequently, the Editorial Board has lost confidence in the reliability of the findings and has decided to retract this publication, as per MDPI’s retraction policy (https://www.mdpi.com/ethics#_bookmark30, accessed on 3 December 2025).

This retraction was approved by the Editor-in-Chief of the journal *Pharmaceuticals*.

The authors disagreed to this retraction.
